# Sarcoglycans are enriched at the neuromuscular junction in a nerve-dependent manner

**DOI:** 10.1038/s41419-025-07353-1

**Published:** 2025-01-22

**Authors:** Michela Gloriani, Bianca Cheli, Chiara D’Ercole, Veronica Ruggieri, Marianna Cosentino, Mireia Serrat Pineda, Biliana Lozanoska-Ochser, Francesca Grassi, Marina Bouché, Luca Madaro, Carles Sánchez Riera

**Affiliations:** 1 Department of Anatomical, Histological, Forensic Sciences and Orthopedics, Sapienza University of Rome, 00161 Rome, Italy; 2 Sorbonne Université, INSERM UMRS 974, Association Institut de Myologie, Centre de recherche en Myologie, 75013 Paris, France; 3 Department of Medicine & Surgery, LUM University, Casamassima, Italy; 4 Department of Physiology and Pharmacology “Vittorio Erspamer”, Sapienza University of Rome, 00185 Rome, Italy

**Keywords:** Synaptic development, Neurodegeneration

## Abstract

Sarcoglycanopathies are heterogeneous proximo-distal diseases presenting severe muscle alterations. Although there are 6 different sarcoglycan isoforms, sarcoglycanopathies are caused exclusively by mutations in genes coding for one of the four sarcoglycan transmembrane proteins (alpha, beta, gamma and delta) forming the sarcoglycan complex (SGC) in skeletal and cardiac muscle. Little is known about the different roles of the SGC beyond the dystrophin glycoprotein complex (DGC) structural role. Here, we show that SGC proteins are enriched at the post-synaptic membrane of neuromuscular junctions (NMJs). Using a mouse model lacking the beta-sarcoglycan subunit, we describe for the first time that the loss of the SGC in the NMJ area results in alterations of pre- and postsynaptic membrane, as well as a significant reduction of membrane potential. Moreover, using different denervated wild-type mouse models, we demonstrate that nerve presence precedes the sarcoglycan enrichment at NMJ, suggesting a nerve-dependent sarcoglycan expression. Altogether, our findings suggest that pathological decline should no longer be understood only in terms of sarcolemma damage but also in terms of sarcoglycans’ participation in the NMJ. Henceforth, our work paves the way for the identification of new mechanisms involving sarcoglycans and new approaches for the treatment of sarcoglycanopathies.

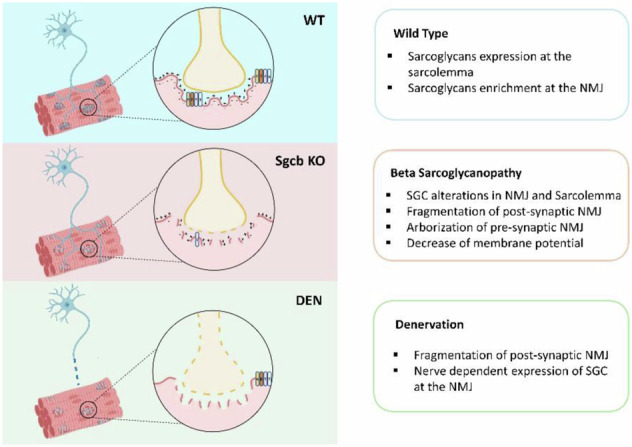

## Introduction

Limb Girdle Muscular Dystrophy (LGMD) types R3, R4, R5, and R6, collectively known as sarcoglycanopathies, encompass a spectrum of genetic disorders characterized by significant heterogeneity that leads to progressive muscle weakness and reduced life expectancy [1–4]. Currently, there is no cure for sarcoglycanopathies [5].

These conditions arise from mutations in genes encoding one of the four subunits of the sarcoglycan complex (α, β, γ, and δ), critical components of the dystrophin-associated glycoprotein complex (DGC) [6–8]. Loss or dysfunction of any of the sarcoglycan subunits jeopardizes the whole sarcoglycan complex [9], rendering muscle fibers susceptible to damage and degeneration. However, there are conflicting data demonstrating that some residual subunits may exist in some patients with mutations in gamma or alpha sarcoglycan genes, indicating that incomplete complexes could be found in the fiber membranes [10, 11]. However, functional studies for gene transfer preclinical studies using the beta-sarcoglycanopathy mouse model have shown a reduction of tetanic force and increased fatigability due to the loss of beta-sarcoglycan gene expression [12, 13].

The primary role of the sarcoglycan complex is maintaining sarcolemma integrity within the DGC. However, the enormous heterogeneity observed among patients and even within muscles of the same patient suggests its involvement in other cellular processes [14, 15]. In accordance with this argument, various proteins of the DGC, such as dystroglycan, dystrobrevin, syntrophin, and even dystrophin, have been found to have non-mechanical roles, including activation of nitric oxide pathways [16], and maturation and maintenance of the neuromuscular junction (NMJ) [17–20].

A prominent feature of the neuromuscular junction is the dense clustering of acetylcholine receptors (AChRs) at the postsynaptic membrane [21]. Glutamate NMDA receptors are also present in the NMJ [22, 23] and colocalize with nNOS [24], which in turn is bound to the dystrophin-glycoprotein complex. In embryonic stages, AChRs are initially distributed evenly across the membrane of the newly formed fibers. With innervation, the pentameric receptor aggregates at sites of nerve contact and stabilize; replacing the gamma subunit by the epsilon subunit [25–27]. This nerve-induced process involves multiple molecules and signaling pathways such as Agrin-Lrp4-Musk pathway which includes Agrin, Lrp4, Musk, Dock7, and Rapsyn [28, 29]. On the other hand, NMDA receptors activated by motor neuron-released glutamate, contribute to synapse maturation by regulating the process of synapse elimination during the first 2 weeks of postnatal life [23]. Possibly, NMDA receptors also play a role in functional adaptations of the adult NMJ [30]. The maturation-clusterization process can also be observed in adult muscle due to the daily turnover of the fibers or after acute damage [31]. Following acute damage, muscle fibers undergo regeneration and this process involves a meticulous net of signals, with the final aim to restore the muscle homeostasis, and to reinnervate the newly formed muscle fibers [32]. When the process of fiber degeneration /regeneration becomes chronic, muscle fibers lose their ability to contrast the damage, starting a process of degeneration and atrophy [31, 33, 34]. Interestingly, accumulating evidence indicates that the nerve-muscle crosstalk may be bidirectional with muscle damage contributing to nerve transmission decline, and nerve damage contributing to muscle atrophy [35]. However, our knowledge of NMJ alterations during chronic muscle diseases comes mainly from the mdx model, where Dystrophin has been observed at the muscle endplate, in particular in the depths of the junctional folds, where it binds voltage-gated Na+ channels and determines the length of synaptic folds [36]. The NMJ’s dystrophin is directly related to other proteins of the DGC such as alpha-dystrobrevin [20], syntrophins [37] or beta-dystroglycan [38] and therefore, alterations in dystrophin causes different degree of junctional fold aberrations [26]. As a result of that, these mouse models present severe NMJ alterations such as arborization of nerve terminal, fragmentation of postsynaptic membrane and transmission failure exacerbate muscular decline [39–41]. Regarding sarcoglycans, only a few early studies have suggested that these proteins may be located at the NMJ [42, 43], but there is paucity of data regarding the impact of sarcoglycans deficiency on neuromuscular communication. Recently Fornetti et al. [44] and Zhao et al. [45], have established a link between alpha-sarcoglycan and the NMJ, pointing to the importance of this complex for nerve-muscle dialog. In this work we investigated the presence of sarcoglycans around the AChR-covered area. Moreover, we investigated whether SGC disruption alters the NMJ structure and whether nerve transmission interruption could disrupt the SGC signal. This study aimed to expand our knowledge of the sarcoglycan complex and its functions in muscle. We lay the foundation for future studies of sarcoglycans’ involvement in other molecular pathways, which could help us better explain the pathophysiology and phenotypic diversity among patients or among muscles of the same person affected by sarcoglycanopathy.

## Results

### Sarcoglycan subunits are present at the NMJ

**Fig. 1:**
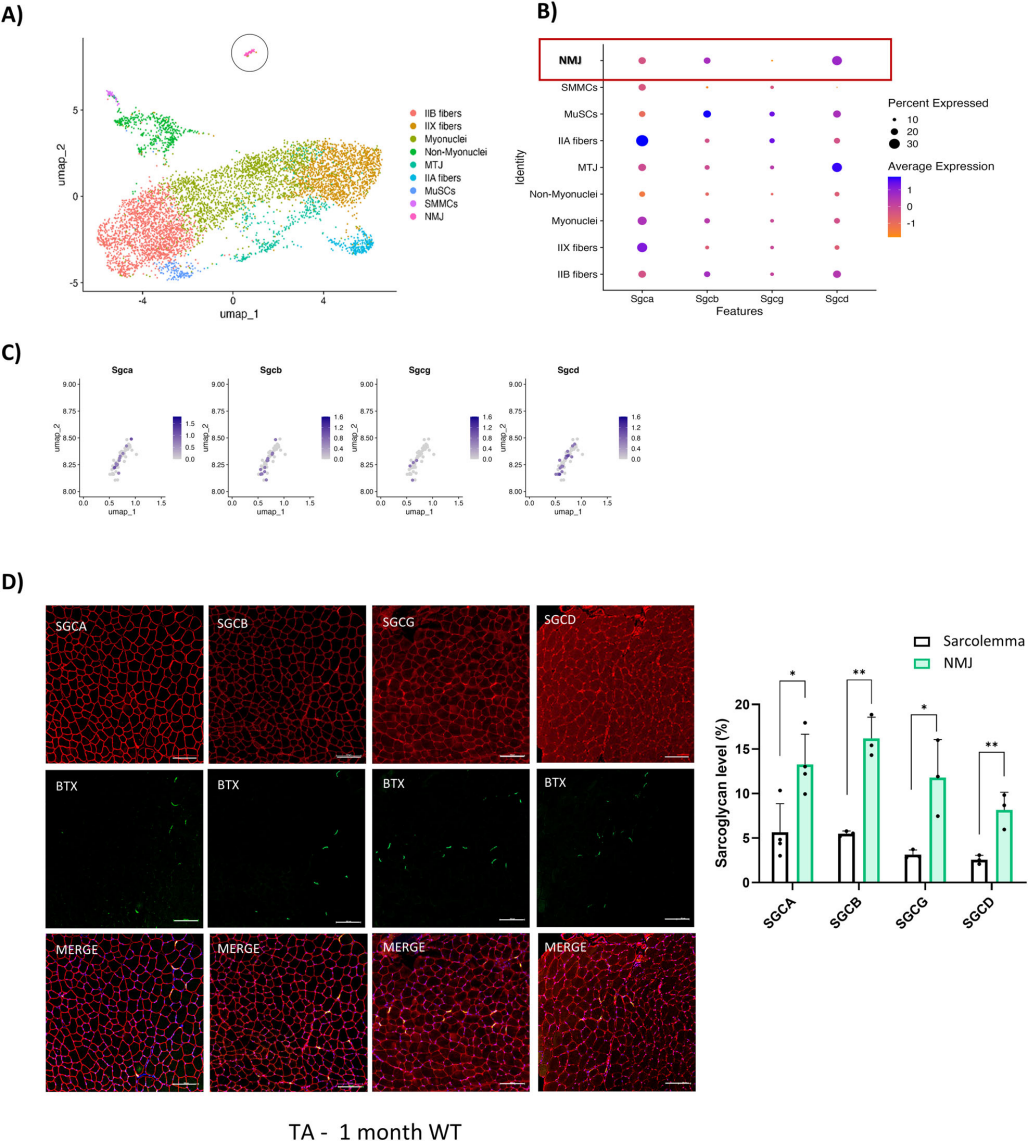
Sarcoglycan subunits are present in the NMJ. **A** Dotplot map of clustered nuclei from snRNA-seq of Petrany et al. [46]. Subsynaptic nuclei represented inside a circle; pink dots. **B** Representation of sarcoglycans average expression level on the different analyzed clusters. **C** Magnification of dotplot map of sarcoglycans relative expression level in NMJ nuclei. **D** On the left, immunofluorescent representative images of Tibialis Anterior transversal sections from WT female at 1 month. Stain of sarcoglycans (red), AChR (green) and dapi (blue). Scale bar 100 μm. On the right, quantification of sarcoglycan signal in identical area size corresponding to sarcolemma section or NMJ section of the immunofluorescence images. *n* = 3. Analysis was made using *t*-test, *p < 0.05; **p < 0.01; ***p < 0.001.

To understand whether sarcoglycan subunits participate in the NMJ, we decided to search for their presence in available online databases. We re-analyzed the Millay’s lab MyoAtlas dataset (https://research.cchmc.org/myoatlas/), which provides single-nucleus skeletal muscle gene expression data [46]. We observed RNA expression of the four sarcoglycan subunits (alpha, beta, gamma, and delta), in nuclei belonging to the neuromuscular junction cluster (Fig. 1A–C). Next, we analyzed the sarcoglycan protein signal along the sarcolemma (Fig. 1D). We identify an enrichment of the four sarcoglycan subunits concomitant to alpha-bungarotoxin for all analyzed muscles; TA (Fig. 1D), diaphragm and triceps (Supplementary Fig. 1A).

### The lack of beta-sarcoglycan alters NMJ morphology

**Fig. 2:**
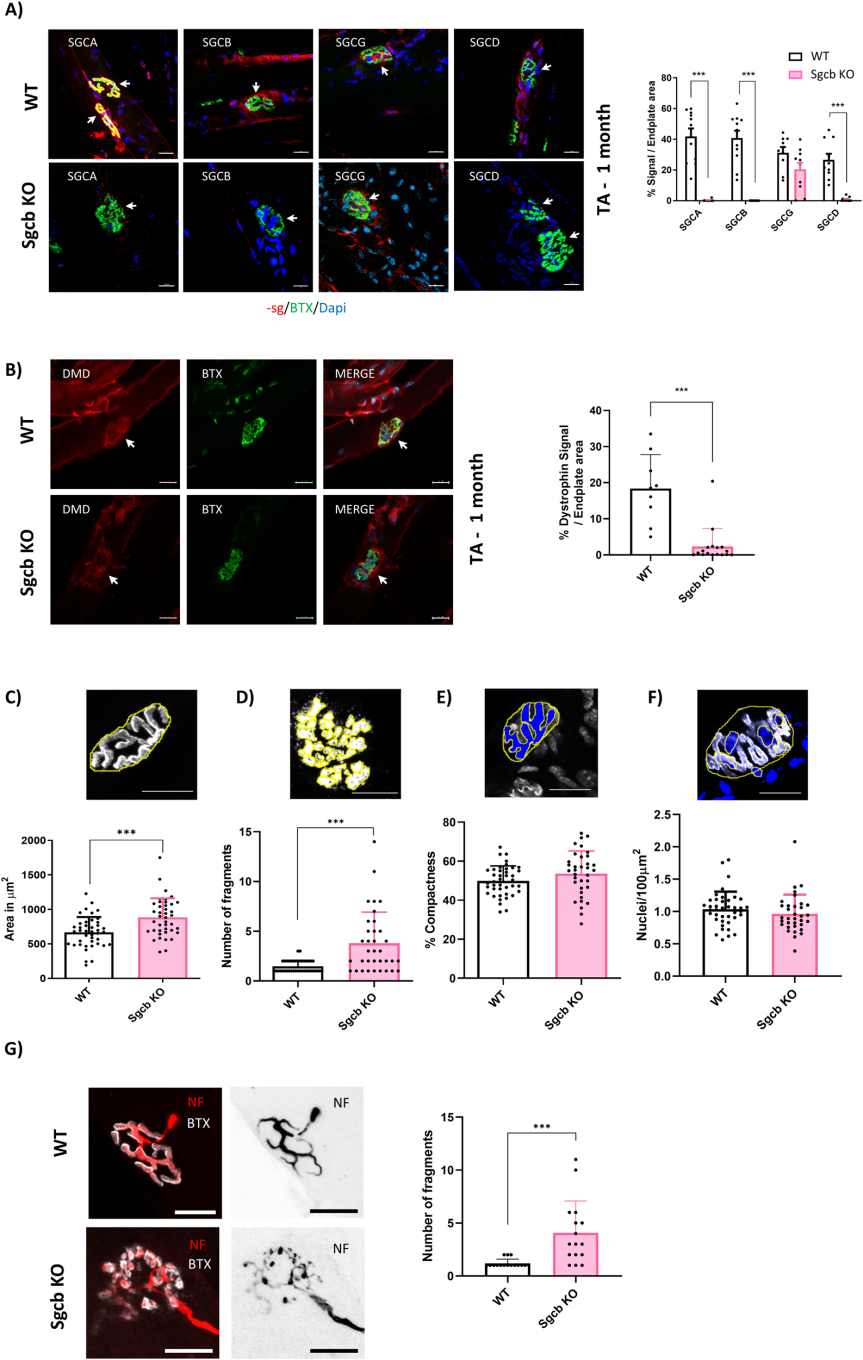
The lack of beta sarcoglycan alters NMJ morphology. All data presented is obtained from female’s Tibialis Anterior muscles at 1 month. **A** Representative whole mount immunofluorescence preparations of fibers stained for sarcoglycan subunits alpha, beta, gamma and delta (red), BTX (green) and dapi (blue) in WT and Sgcb KO muscles. Sarcoglycans on NMJ are evidenced with white arrows. Scale bar 20 μm. On the right, histogram of sarcoglycan subunits signal in endplate area. *n* = 9. **B** Representative whole mount immunofluorescences of fibers stained for dystrophyn (red), BTX (green) and dapi (blue) in WT and Sgcb KO muscles. Dystrophyn on NMJ are evidencied with white arrows. Scale bar 20 μm. On the right, histogram of dystrophin signal in endplate area. *n* = 9. **C**–**F** On the top, schematic representation, on the bottom morphometric measurements of; endplate area (**C**), fragmentation (**D**), compactness (**E**) defined as AChR area/Endplate area and Nuclei/area (**F**). *n* = 30. **G** Representative whole mount immunofluorescences of fibers stained for neurofilament (red), BTX (white) or neurofilament alone on the right (black). On the right, histogram of fragments per terminal. *n* = 15. All analyses were made using *t*-test, *p < 0.05; **p < 0.01; ***p < 0.001.

We performed whole mount immunofluorescence on isolated WT fibers to better understand the location of sarcoglycan subunits at the NMJ. As shown in Fig. 2A, protein localization in the NMJ was observed for the four analyzed subunits. Next, using the beta-sarcoglycan KO mouse model [47] we observed a reduced expression or absence of the other sarcoglycan subunits at the NMJ. Notwithstanding, the gamma-sarcoglycan subunit was still detectable at the NMJ despite the lack of beta-sarcoglycan, suggesting its independent clustering dynamics compared to the others (Fig. 2A and Supplementary Fig. 2A). Of note, the AChR remained detectable despite sarcoglycans absence. On the other hand, in WT muscle, the alpha sarcoglycan signal was colocalized with AChR signal while the other subunits are close to AChR signal without overlapping it (Supplement Fig. 2B). Furthermore, we observed a reduction of dystrophin at the NMJ level (Fig. 2B), suggesting that the loss of beta-sarcoglycan may compromise other members of DGC. To further characterize this postsynaptic destabilization in the beta-sarcoglycan KO mouse model, we studied the morphological parameters of AChRs distribution in the endplate. We observed statistically significant increases of endplate area (Fig. 2C) and AChR fragmentation compared to WT (Fig. 2D), but no differences in compactness (Fig. 2E). Contrary to what was described in previous studies [48] we observed a similar amount of subsynaptic nuclei between WT and KO (Fig. 2F).

Additionally, despite no denervation being observed in Sgcb KO muscle sections, as shown by the high rate of colocalization between BTX and neurofilament (Supplementary Fig. 2C) and the upward trend in synaptophysin (Supplement Fig. 2D), axon terminals were deeply affected, showing an aberrant arborized morphology of nerve terminals (Fig. 2G). Altogether, these results demonstrate that the lack of beta-sarcoglycan protein results in morphological destabilization of both pre- and postsynaptic structures.

### The lack of beta-sarcoglycan impacts membrane potential of muscle fibers

**Fig. 3:**
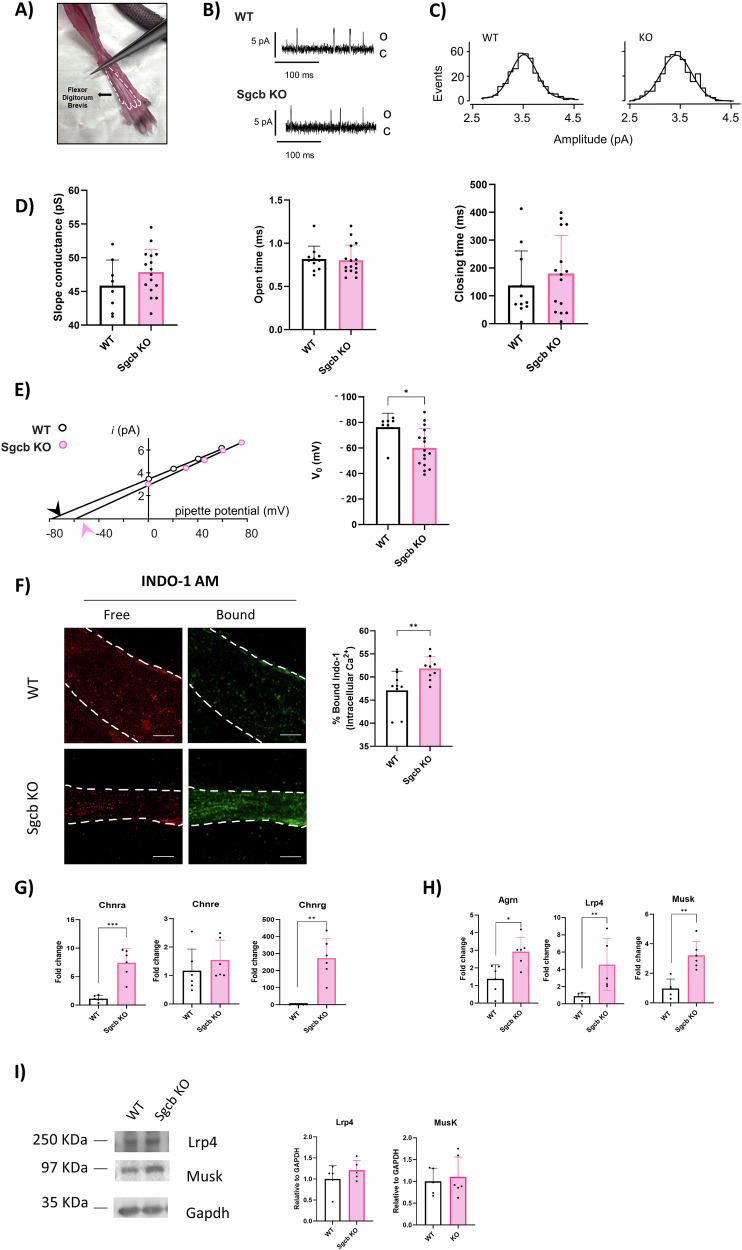
ACh-evoked unitary events in muscle fibers of WT and KO mice. **A** Image of mouse hindlimb with flexor digitorum brevis (FDB) evidenced (white). **B** Representation of typical ACh-evoked unitary events recorded in visually identified NMJ on fibers from mice of the indicated strain. **C** Representative image of histogram of the amplitude of unitary events at 0 mV pipette potential. **D** Average values of slope conductance’s per strain (left), open time (centre) and closed duration (right) of the events of recorded patches. Measurements were made in 8–16 fibers as indicated (3 WT, 5 Sgcb KO mice). **E** Plot of the unitary amplitude *vs*. pipette potential in the same single fibers, with the best fitting lines. Arrowheads represent the reversal potentials (V_0_). On the right, histogram of average measurements of reversal potentials. **F** Representative images of Indo immunofluorescence on WT and Sgcb KO female fibers; free Indo (red) and bound Indo (green), the fibers are outlined with a white dashed line. On the right, histogram relative to Indo-1 percentage. *n* = 9. **G** RNA levels of AchR subunits alpha (left), gamma (centre) and epsilon (right) on WT and Sgcb KO muscles. *n* = 6. **H** RNA levels of Agrin pathway; Lrp4 (left), Agrin (centre) and Musk (right) on WT and Sgcb KO muscles. *n* = 5. **I** Western blot representative images of Lrp4 and Musk. Gapdh was used as internal control. On the right, histogram with WB samples quantification. *n* = 5. All analyses were made using *t*-test, *p < 0.05; **p < 0.01; ***p < 0.001.

To understand whether morphological changes in the NMJ could be associated to changes in AChR functionality, we recorded ACh-evoked channel activity on WT and Sgcb KO muscles. We used isolated fibers of flexor digitorum brevis (FDB), a glycolytic fast twitch distal muscle from 4 to 8 weeks old mice (Fig. 3A). Synaptic regions were visually identified, and openings of synaptic AChR-channels were characterized by the channel conductance, open duration and closed time of unitary events. In both strains, openings of only one channel population were detected (Fig. 3B, C). All functional parameters were similar in WT and KO fibers (Fig. 3D) and compatible with the openings of AChR-channels containing the epsilon subunit [27]. In several fibers, recordings were also performed in non-synaptic regions, observing only occasional openings of AChR-channels (data not shown). These functional observations indicate that fibers were not denervated. However, the plot of unitary current of each single fiber vs. pipette potential showed a statistically significant reduction of the current reversal potential in Sgcb KO fibers (Fig. 3E).

Additionally, we found that the level of intracellular Ca2+ in another distal muscle such as TA, was slightly but significantly increased in Sgcb KO mouse fibers (Fig. 3F) despite non-significant differences in the amount of damaged fibers and inflammation markers were observed histologically (Supplementary Fig. 3A, B). On the other hand, upregulation of AChR transcription levels was detected (Fig. 3G). We observed a significant increase of the alpha and gamma AChR subunits compared to WT. By contrast, the transcript level of the epsilon subunit, replacing the gamma subunit in adult innervated fibers, was similar to WT (Fig. 3G). Of note, based on patch-clamp experiments, no opening of AChR-channels containing the gamma subunit were detected in synaptic or extra-synaptic regions. In any case, the upregulation of AChRs transcripts was concomitant with a transcriptional upregulation of Agrin-Lrp4-Musk pathway (Fig. 3H), not shown at protein level as revealed by Lrp4 and Musk (Fig. 3I). These results were observed together with an upward trend in acetylcholinesterase transcription (Supplementary Fig. 3C) but not of choline acetyltransferase protein level (Supplementary Fig. 3D). The upregulation pattern in Sgcb KO conditions was not observed during regeneration process after acute damage (Supplementary Fig. 3E, F).

### Nerve damage disrupts the SGC at the NMJ

**Fig. 4:**
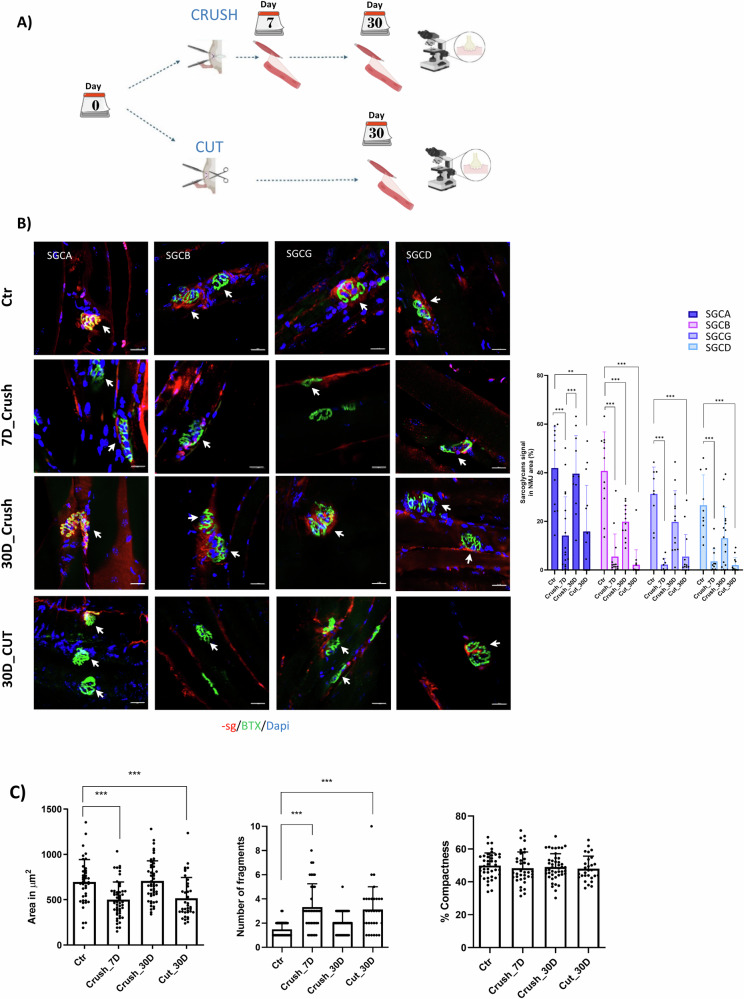
Nerve damage disrupts SGC in NMJ. Experiment realized on TA muscles of WT female mice at the ages of 4–8 weeks. **A** Scheme of the experiment showing the kind of sciatic nerve damage and the timeline of experimental points after the crush or cut. **B** On the left, representative images of immunofluorescence whole mount TA fibers at 0 days (control), 7 days after crush injury (denervated), 30 days after crush injury (reinnervated) and 30 days after cut injury (denervation). NMJ zone is evidenced with white arrows. The staining was made with antibodies against each sarcoglycan subunits (red), BTX (green) and dapi (blue). Scale bar 20 μm. On the right, histogram of sarcoglycan subunits at endplate area associated to 4B images. *n* = 8. **C** Histograms of morphologic parameters; endplate area (left), fragmentation (centre) and compactness (right) on WT and Sgcb KO fibers. *n* = 17. Analyses were made using One-way ANOVA, *p < 0.05; **p < 0.01; ***p < 0.001.

To further understand the relation of sarcoglycans with NMJ structure and function, we investigated whether sarcoglycan enrichment within the NMJ structure was altered upon perturbations or damage to the nerve. We used two different mouse models of nerve damage: sciatic nerve crush and sciatic nerve cut (Fig. 4A), which allowed us to simulate and compare reversible and chronic damage, respectively [33, 34, 49]. We analyzed sarcoglycan expression and distribution by whole-mount immunofluorescences at time 0 (before the damage), at 7 and 30 days after nerve crush, or 30 days after nerve cut (Fig. 4A). In both cases, we observed a severe reduction of sarcoglycan subunits when the muscle was denervated: 7 days after crush or 30 days after cut (Fig. 4B). The signal was reestablished to basal levels 30 days after crush when the muscle was reinnervated. No alterations were observed in the contralateral legs (Supplementary Fig. 4A). Along with the loss of sarcoglycan subunits, we observed reduced endplate size and increased AChR signal fragmentation but not changes in compactness (Fig. 4C). Interestingly, the expression of the gamma sarcoglycan subunit, which retained some level of expression in the absence of beta-sarcoglycan (Fig. 2A and Supplementary Fig. 2A), was dependent on nerve signals, as were the other subunits. We observed no changes on morphological parameters at the contralateral leg (Supplementary Fig. 4B). Altogether, these results suggest that the enrichment of the sarcoglycan complex at the NMJ is dependent on nerve signals.

### Sarcoglycans enrichment at the NMJ is secondary to AChRs-spot formation

**Fig. 5:**
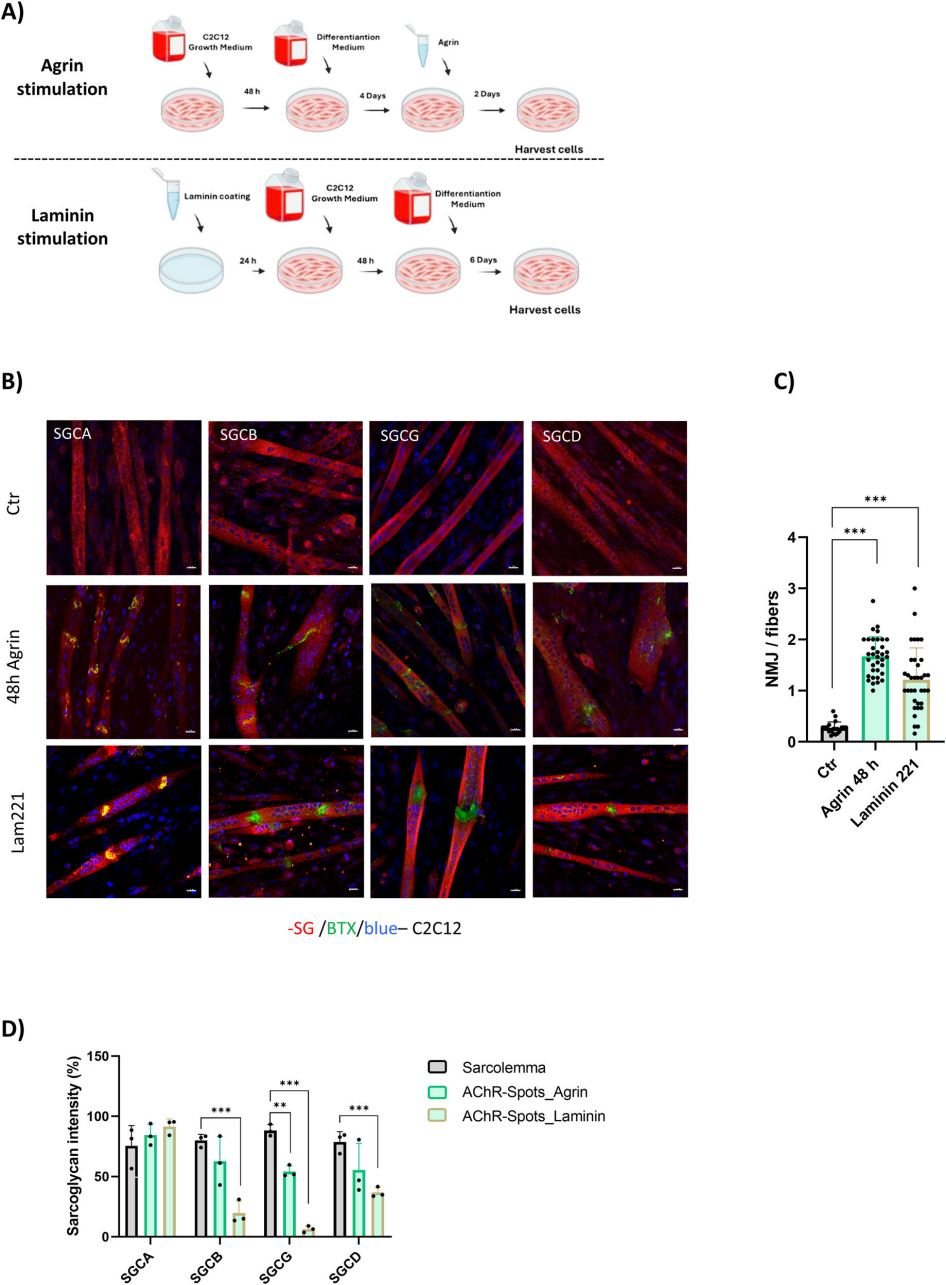
Sarcoglycans enrichment at the NMJ is secondary to AChRs-spot formation. In vitro experiments conducted with c2c12 p8. **A** Scheme of the experiment showing the time line of the critical points, to get myotubes with AChR-spots after Agrin stimulation or Laminin stimulation. **B** Immunofluorescence of c2c12 stained with antibodies against sarcoglycan subunits (red), BTX (green) and dapi (blue). Scale bar 20 μm. **C** Histogram of AChR-spots per fiber after the stimulation or not with Agrin or Laminin. *n* = 25. **D** Histogram of sarcoglycan subunits intensity on sarcolemma (gray columns), AChR-spots area of Agrin stimulated myotubes (green columns) and AChR-spots area of Laminin stimulated myotubes (gold-green columns). *n* = 3. All analyses were made using One-way ANOVA, *p < 0.05; **p < 0.01; ***p < 0.001.

To further investigate whether sarcoglycan enrichment at NMJs is due to the nerve presence, we used an in vitro system. C2C12 cells were cultured and differentiated into myotubes (Fig. 5A). We then induced AChR-spot formation in myotubes, treating them with agrin or laminin (Fig. 5B) [50]. As expected, an increase in AChR-spot formation was observed after treatment in both cases (Fig. 5C). In agrin stimulated myotubes, the levels of sarcoglycan subunits at the sarcolemma and in AChR-spots was similar, with the exception of gamma subunit that was widely downregulated (Fig. 5D). On the other hand, myotubes stimulated with human recombinant laminin showed a dramatic reduction of sarcoglycan subunits in the areas of AChR-Spot formation compared to the sarcolemma of the same fibers. An exception was alpha-sarcoglycan which showed some colocalization (Fig. 5D). Altogether, these results indicate that no enrichment of sarcoglycan subunits at the AChRs clustering area occurs in the absence of nerve.

## Discussion

Among sarcoglycans, alpha, beta, gamma and delta are transmembrane proteins mainly expressed in skeletal and cardiac muscle fibers. Even though their main role has been primarily related to support of the sarcolemma within the DGC, the expression of some subunits has also been linked to the NMJ [44, 45]. However, no studies have characterized their presence as a complex in the postsynaptic structure until now.

In this study, we show for the first time that the entire SGC is enriched at the NMJ. We observed that the absence of the complex alters the morphology of pre and postsynaptic membranes in the NMJ and provokes a reduction of the membrane potential even in distal less affected muscles. Finally, using different models of denervation we provide evidences that sarcoglycans enrichment specifically requires nerve-derived signals other than those required for the formation of AChR cluster, suggesting a possible participation of sarcoglycans to unknown specific role(s) related to nerve signal transmission.

First, on WT mouse we observed an enrichment of sarcoglycan signal corresponding to the AChR clusterization sites. Furthermore, using a Sgcb KO mouse model, we observed differences between the SGC in the sarcolemma and the SGC at the NMJ. Whereas in the sarcolemma, the loss of beta sarcoglycan subunit results in the loss of all subunits, at the NMJ, the gamma subunit expression was preserved (Fig. 2A). These observations are compatible with the hypothesis of Draviam et al. [51], who found that alpha-sarcoglycan moves alone to the sarcolemma with incomplete glycosylation state, where it joins the beta-delta core giving stability to the complex. Draviam et al. found incomplete complexes at different cell structures and the complete tetramer just at the sarcolemma. We hypothesize that beta-delta-alpha, without gamma subunit, may find stability among NMJ proteins such as Lrp4 [45]. Observations of no defects in NMJ in the gamma-sarcoglycan KO mouse model [52], further support the idea that the gamma subunit may move to NMJ using alternative pathways. Another hypothesis, could be that the gamma subunit is alternatively substituted with the zeta subunit as it happens at the smooth muscle coronary arteries [53]. Next, in the absence of SGC induced by beta-sarcoglycan deficiency, we observed morphological changes in pre and postsynaptic elements of the NMJ, including arborization of nerve terminal and enlargement and fragmentation of postsynaptic membrane. These findings, reported for the first time in sarcoglycanopathies, have been previously described in mdx mice [48, 54] and in chimeric mice lacking dystroglycans [38]. Indeed, the lack of sarcoglycans at the NMJ was followed by a decrease of dystrophin protein in the endplate area. Both down regulations suggest to us that other proteins of DGC may also be dysregulated. However, it remains unclear whether they are a secondary effect of sarcolemma damage or a direct effect of the degeneration/regeneration process [55]. In spite of limited structural damage, we report depolarization of resting membrane potential and increased levels of intracellular Ca2 + , possibly secondary to sarcolemma damage. On the other hand, we observed increased transcriptional levels of some AChR subunits together with increased levels of the Agrin-Lrp4-MuSK pathway components and AChE, but no changes in acetylcholine transferase (ChAT) protein presence. These findings, observed in mdx as well [41], may suggests an activation of the regeneration process in Sgcb KO muscles, although we observed no upregulation of AChR subunits or Agrin-Lrp4-MuSK pathway during regeneration process after Ctx injection (Supplementary Fig. 3E, F). Therefore, we believe that one possible explanation of the AChR upregulation could lie in some qualitative compensatory mechanism related to the subsynaptic nuclei accumulated at the endplate area. Although WT and KO show similar numbers of subsynaptic nuclei, further investigations should address whether they have specific gene signature.

To further investigate whether nerve signals were triggering SGC accumulation in the NMJ, we conducted experiments in WT mice with partial or total denervation of the sciatic nerve and in vitro experiments. From these experiments, we observed that after nerve crush or cut, sarcoglycans disappeared from the postsynaptic part of the NMJ, but persisted in the sarcolemma. These findings, together with our in vitro experiments performed in the absence of nerve where no accumulation of sarcoglycans in the area of AChR spots occurred, identify the nerve as an essential upstream element for sarcoglycan complex enrichment at the NMJ.

Despite several limitations, such as not being able to identify the type of subsynaptic cells expressing enhanced levels of sarcoglycans or whether the arborization of the nerve terminal is related to declined neurotransmission, our study suggests an unknown nerve-dependent link between the nerve signal and the SGC. According to our experiments and the data published from Fornetti [44] and Zhao [45] we hypothesize that the alpha subunit may be the first to be attached to the NMJ postsynaptic area, where the sarcoglycan beta-delta core join and stabilize the complex. We speculate that SGC may has a role in the signal transmission. One possible mechanism of action may be related to the alpha-sarcoglycan ATP-hydrolyzing activity demonstrated by Dr. Sandonà and colleagues [56, 57]. As ATP is the main co-transmitter release by nerve with acetylcoline [58], we believe that alpha-sarcoglycan hydrolyzing activity at NMJ may contribute to conformational changes on the SGC, possibly initiating an unknown molecular pathway. Other mechanisms to be validated may be in line with recent findings on NMJ maturation and maintenance [22, 23, 30] and the glutamate contribution to SGC complex stabilization in the endplate area.

The participation of sarcoglycans in nerve transmission may be among the reason why muscle pathologies, and sarcoglycanopathies in particular, commonly increase their degeneration rates even without mechanical injury [17] and especially, after patients loss of ambulation [15]. However, further research is needed to unravel the possible participation of the sarcoglycan complex at the NMJ transduction signal and its involvement in new molecular pathways. The multiple glycosylation and phosphorylation sites of all sarcoglycan subunits [59] might help to validate or to refute some hypotheses regarding sarcoglycan localization or conformational state respectively.

In conclusion, our work opens new avenues for exploring the role of SGC in the NMJ and its contribution to sarcoglycanopathies focusing on the nerve’s influence on muscle disease.

Understanding the specific role of the sarcoglycan complex at the NMJ could lead to innovative therapeutic approaches targeting this critical synaptic interface.

## Materials and methods

### Bioinformatic analysis

The post-natal (P) 21 day WT dataset from Petrany and colleagues [46] was obtained by subsetting the fully processed scMuscle compendium Seurat object from McKellar et al., available for download on Dryad [60]. Data were normalized using Seurat’s SCTransform function (Seurat v5) [61]. Dimensionality reduction was performed through Principal Component Analysis (PCA) on top variable features, with npcs = 30. FindNeighbors, FindClusters, and UMAP embedding functions were based on the top 30 PCs (Principal Components) of the PCA. We identified 12 clusters using FindClusters with parameters resolution = 0.9. After clustering, cell populations were annotated using the “FindAllMarkers” function according to well-known lineage markers, resulting in 9 meta-clusters. The annotated dataset was used to generate the dotplot in Fig. 1B and the FeaturePlot in Fig. 1C.

### Mice

WT C57BL/6 J mice and B6.129-Sgcbtm1Kcam/2 J mice (Sgcb KO, RRID:IMSR_JAX:006833) were purchased from Jackson Laboratory. KO mice were bred and maintained as homozygous animals in standardized conditions. Since no gender differences regarding muscle pathology have been described on these mice [47], we performed experiments on females to minimize genetic drift.

### Ethics approval and consent

All experiments in this study were conducted in accordance with protocols approved by the Italian Ministry of Health, Rome, Italy, (Autorization n° 707/2023PR) and the ethical regulations of the University of Rome 1 “Sapienza” (Rome, Italy). Informed consent was obtained from all participants.

### Denervation

TA muscles from 4 week-old female WT C57BL/6 J mice were used for this experiment. Briefly, mice were deeply anesthetized with a mixture of Rompun (Bayer, 20 mg/mL; 0.5 mL/kg) and Zoletil (100 mg/mL; 0.5 mL/kg). For crush injuries, the sciatic nerve was compressed at three different points for 5 s. For cut injuries, ~3 mm of the sciatic nerve was removed, followed by wound closure with tissue adhesive (Vetbond). Buprenorphine (0.05–0.1 mg/kg) was administered subcutaneously prior to the animals regaining consciousness [34].

### Immunofluorescence

TA muscles from WT C57BL/6 J and Sgcb KO mice females, aged 4–8 weeks, were used to obtain cryosections or whole mount preparations of entire fibers. Cryosections were permeabilized with 100% acetone, while fibers were fixed in 4% PFA (MilliporeSigma, P6148) at room temperature. A blocking solution containing 4% BSA (MilliporeSigma, A7030-100G) was applied to both types of preparations. Primary antibodies were applied overnight at 4 °C: anti-Sgca (Ab189254), anti-Sgcb (Ab315386), anti-Sgcg (Nova Castra 6091787), and anti-Sgcd (kindly provided by Dr. Sandonà). Secondary antibodies goat anti-rabbit Alexa Fluor 594 (A11011) and goat anti-mouse Alexa Fluor 594 (A21235) were then applied. Alpha-Bungarotoxin coupled to Alexa Fluor 488 (B13422) was used to identify AChR presence. Counterstaining was performed with DAPI (Thermo Fisher Scientific, D1306). All images were acquired using confocal microscopy with z-stack intervals of 1 µm. Images were subsequently analyzed using ImageJ software.

### Sarcoglycan intensity determination and morphological analysis

The sarcoglycan signal was calculated as the threshold value of sarcoglycans in a determined area. This same area was used to evaluate the signal at the sarcolemma or at the NMJ. Morphological analysis consisted in the assessment of endplate area, fragmentation, compactness and subsynaptic nuclei clusterization. Additionally, colocalization of AChR signal with sarcoglycans was also analysed. All analyses were performed following standard procedures described by Jones and colleagues [62]. Briefly; endplate area was calculated as the area of AChR perimeter at the post-synaptic membrane. Fragmentation; as the number of fragments on the endplate area. Compactness, as the ratio between the AChR area and endplate area. Subsynaptic nuclei, as the number of nuclei on the endplate area.

### Muscle fiber preparation and cell-attached patch-clamp recordings

Flexor digitorum brevis (FDB) muscles from WT and Sgcb KO mice were dissected from the hindlimbs of female mice aged 4–8 weeks, which were sacrificed by cervical dislocation. The FDB muscles were incubated with Type I collagenase (3 mg/mL, Sigma) for 30 min at 37 °C in Minimum Essential Medium (MEM) (Gibco, Thermo Fisher Scientific, #11095-080). After equilibrating in Ca2+ free suspension culture MEM (Gibco, #11380-037) for 30 min at room temperature, the muscles were gently triturated until to obtain single fibers. Recordings were performed on fibers imaged by a phase-contrast microscope (Axioskop 2 FS, Zeiss, Jena, Germany). Patch pipettes were filled with a solution containing (in mM, all from Sigma Aldrich, St. Louis, MO, USA): NaCl 140, KCl 2.8, CaCl2 2, MgCl2 2, glucose 10, HEPES/NaOH 10, pH 7.3, plus ACh (100 nM). The resistance was 4–6 MΩ. Single-channel currents were recorded at room temperature (23–26 °C) using a low-noise Axo-patch 200B amplifier (Molecular Devices, San Jose, CA, USA) in cell-attached mode. Data were sampled at 25 kHz, digitally filtered at 5 kHz (Gaussian filter), and analyzed using pClamp 9.2 (Molecular Devices). Only single openings (100–3000 at each pipette potential in every patch) were used to determine the channel slope conductance and duration. Measurements were performed at the same pipette potential at the beginning and end of each recording to ensure membrane potential stability. Slope conductance was calculated only if the membrane potential was stable and unitary events were recorded at least at three different pipette potentials. The kinetic properties of ACh-evoked events were compared at an estimated membrane potential of –90 ± 10 mV, assuming a reversal potential of 0 mV. Histograms of open times were fitted with sums of exponential components using a non- linear algorithm (pClamp 9.2), as required.

### RNA extraction and real time PCR

Total RNA was extracted from frozen female tibialis anterior muscles using TRIzol Reagent (Life Technologies, 15596018) following the manufacturer’s protocol. The RNA was then reverse transcribed using the High-Capacity cDNA Reverse Transcription Kit (Life Technologies, 4368814). For quantitative PCR (qPCR), premade TaqMan assays labeled with 6- Carboxyfluorescein (FAM) (Thermo Fisher Scientific) were used.

### Intracellular calcium levels determination

INDO-1AM (Invitrogen, I1226) was reconstituted in high-quality, freshly opened DMSO to a concentration of 1 mM. For experiments, it was used at a final concentration of 100 µM in Ca2 + /Mg2 + -free PBS (phosphate-buffered saline). After reconstitution, the solution was protected from light and stored at −20 °C to prevent freeze-thaw cycles. Samples were washed three times with Ca2 + /Mg2 + -free PBS before slowly adding INDO-1AM along the muscle fibers. The samples were then incubated for 30 min at 37 °C. Following incubation, the samples were washed three times with Ca2 + /Mg2 + -free PBS and analyzed using confocal microscopy. Detection was based on the dual emission properties of INDO-1, which shifts from 475 nm in Ca2 + -free media to 400 nm when saturated with Ca2 + [63]. All analyses were conducted using Zeiss Confocal Software (Zen 3.0 Blue Edition).

### Western blot analysis

Protein extraction and western blotting were carried out using standard procedures [9]. List of primary antibodies is detailed in Supplementary Figure Western Blots.

### In vitro cultures

C2C12 were cultured until differentiation following standard procedures and then, stimulated with agrin (RD Systems, 550-AG) following the procedure described in Proietti et al. [49].

### Statistical analysis

All statistical analyses were conducted using Prism version 9.0 (GraphPad Software, San Diego, CA, USA). Data are presented as the mean ± SD. Statistical significance was assessed using paired Student’s *t*-test, One-way ANOVA, as specified in each figure legend. A *p*-value of <0.05 was considered statistically significant (∗*p* < 0.05, ∗∗*p* < 0.01, ∗∗∗*p* < 0.001), while *p*-values ≥ 0.05 were considered not significant.

## Supplementary information

Supplementary 1

Supplementary 2

Supplementary 3

Supplementary 4

Uncropped WB.

## Data Availability

All data generated is available in the manuscript however, if further information is needed it can be available contacting the corresponding author.
