# Supplementary figures and images for: Sarcoglycans are enriched at the neuromuscular junction in a nerve-dependent manner
Source: Cell Death Dis. 2025 Jan 22;16(1):37. doi: 10.1038/s41419-025-07353-1 (PMC11754441; doi:10.1038/s41419-025-07353-1)

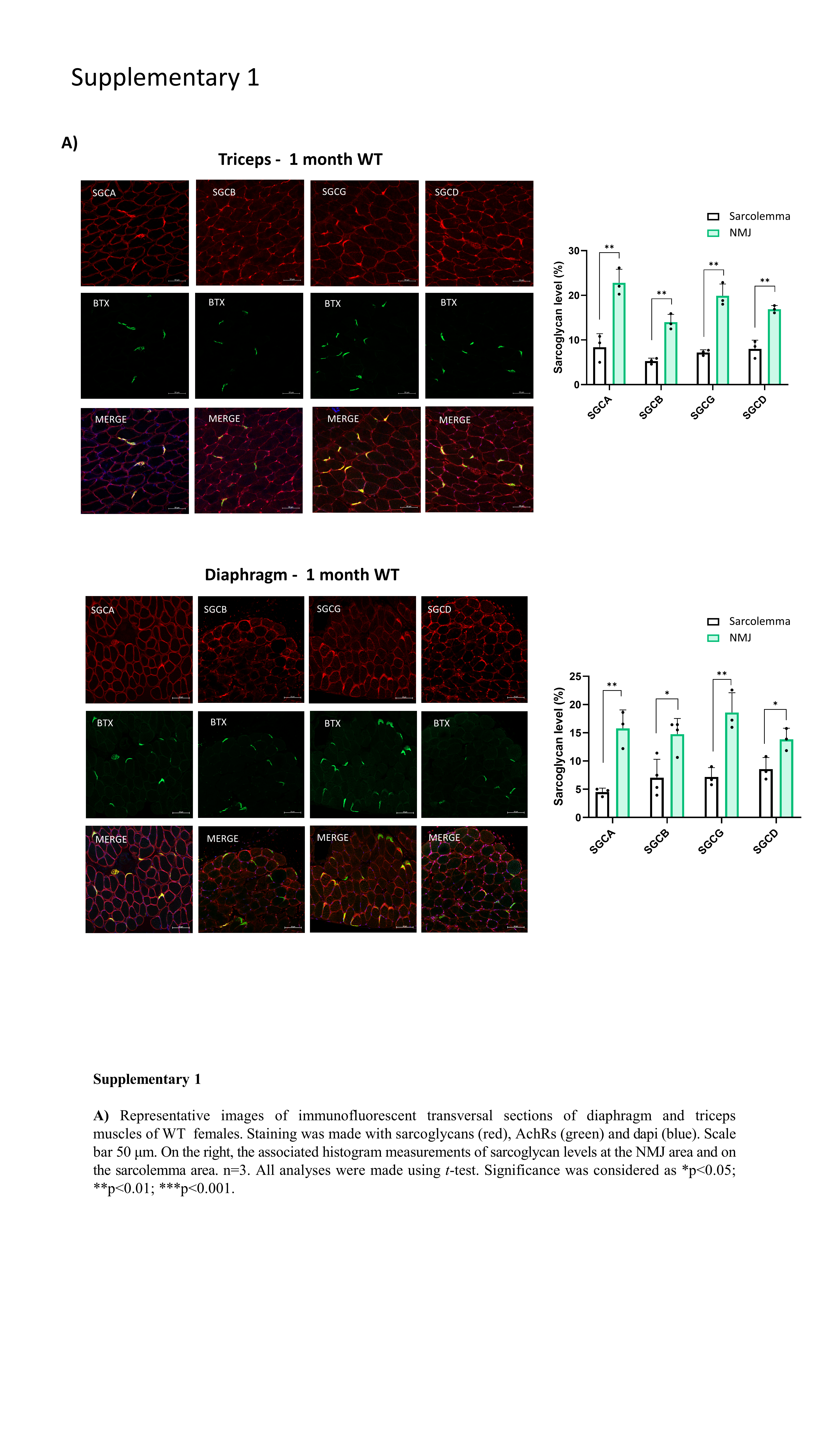

Supplement: Supplementary file 1 — Supplementary 1 [file 41419_2025_7353_MOESM1_ESM.tif]

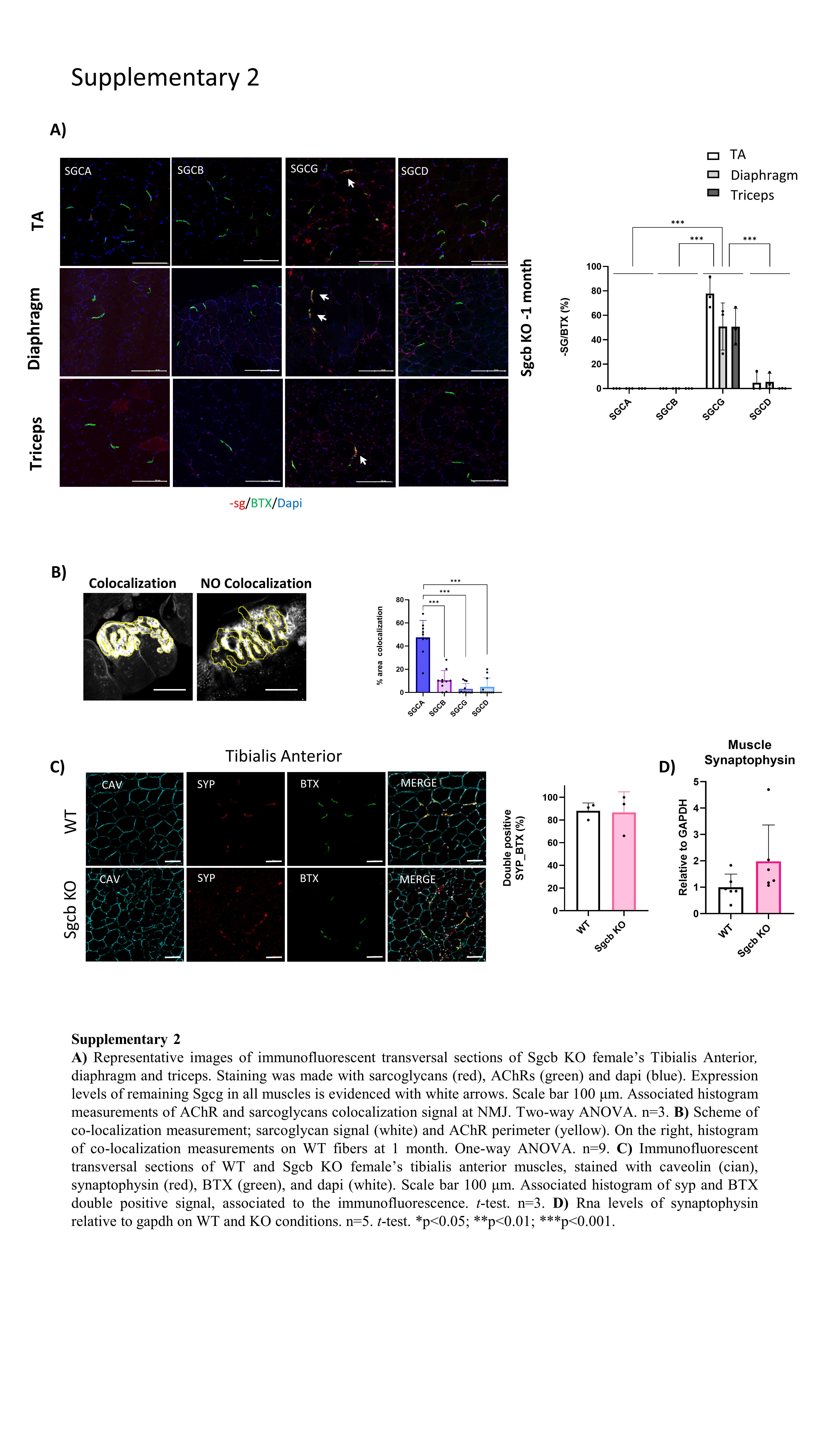

Supplement: Supplementary file 2 — Supplementary 2 [file 41419_2025_7353_MOESM2_ESM.tif]

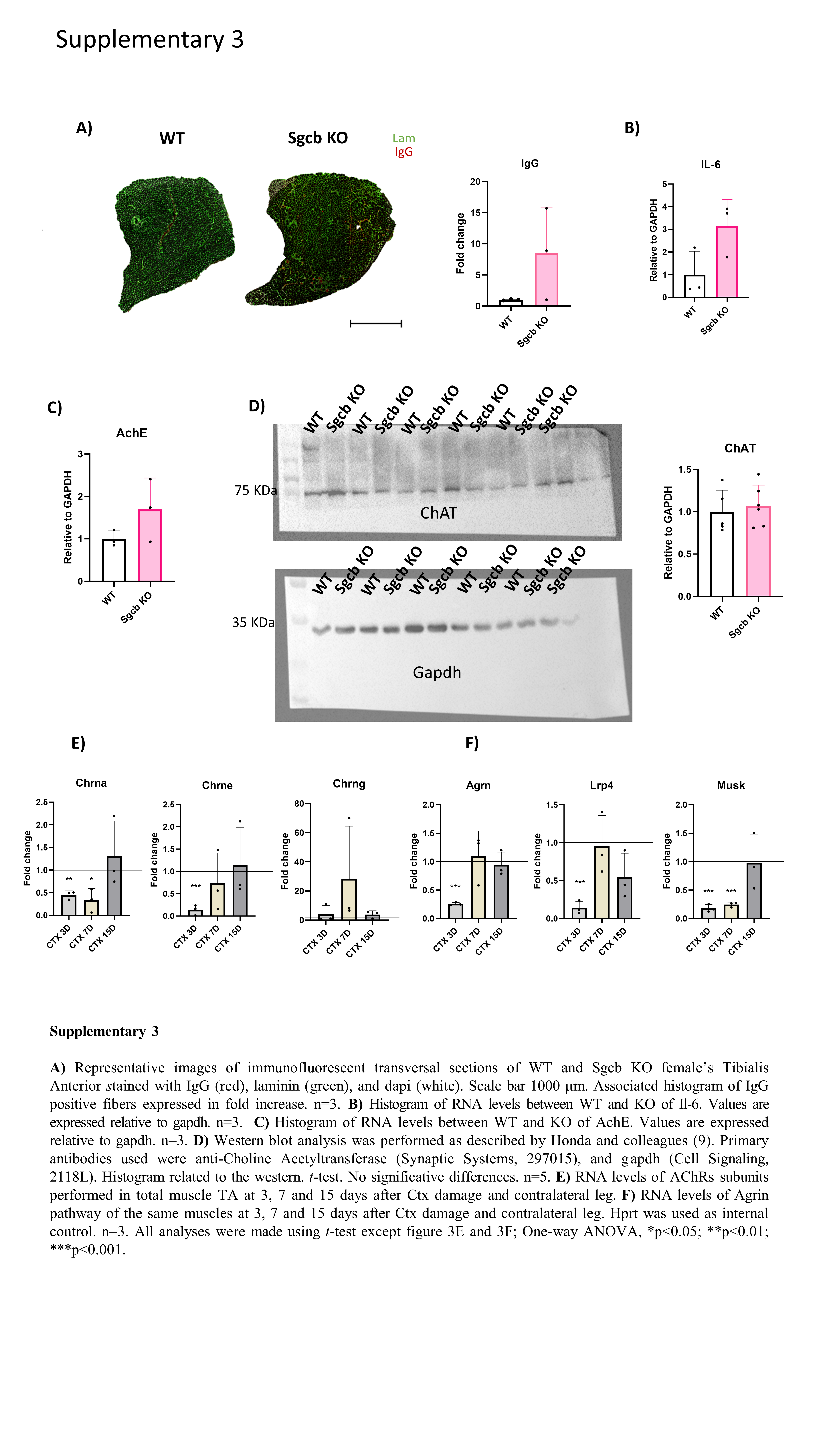

Supplement: Supplementary file 3 — Supplementary 3 [file 41419_2025_7353_MOESM3_ESM.tif]

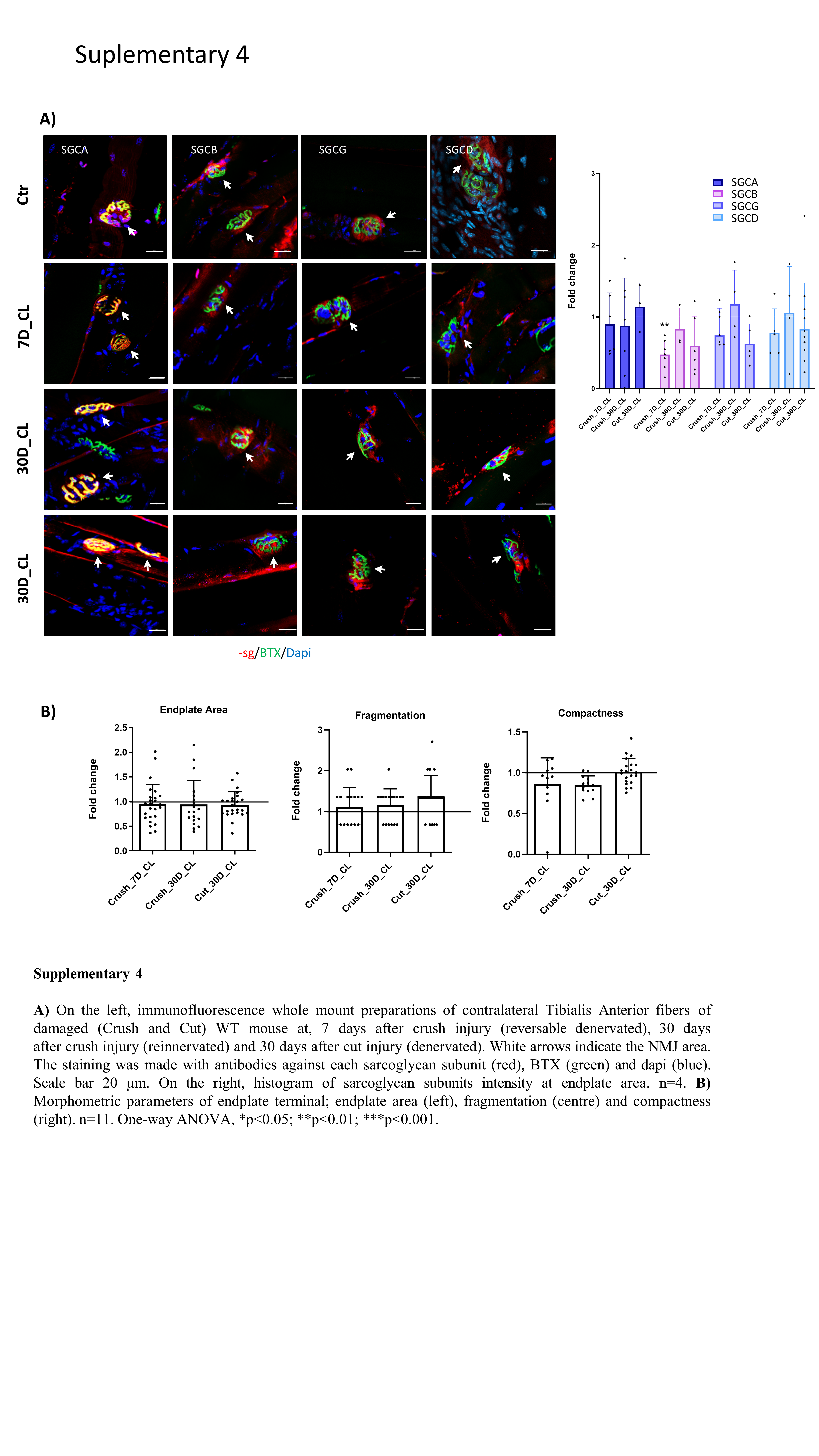

Supplement: Supplementary file 4 — Supplementary 4 [file 41419_2025_7353_MOESM4_ESM.tif]

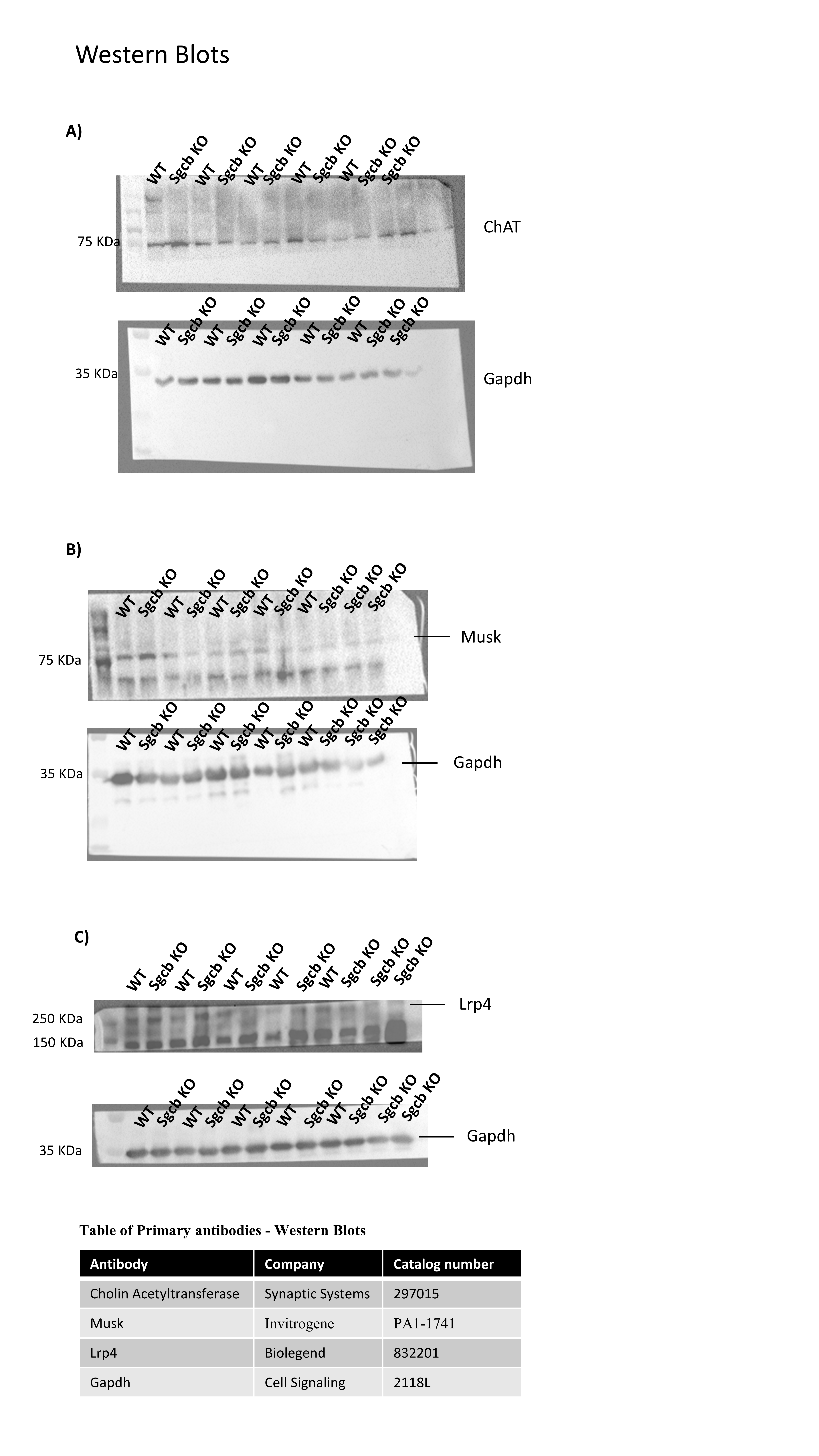

Supplement: Supplementary file 5 — Uncropped WB. [file 41419_2025_7353_MOESM5_ESM.tif]
